# Tumor Disarmed: A Strategic Approach to Cervical Osteoblastoma Near Vital Vessels in a Young Patient

**DOI:** 10.7759/cureus.95113

**Published:** 2025-10-21

**Authors:** Bharat R Dave, Mahesh Sagar, Shivanand C Mayi, Ravi Ranjan Rai, Mirant B Dave, Degulmadi Devanand, Ajay Krishnan

**Affiliations:** 1 Spine Surgery, Stavya Spine Hospital and Research Institute, Ahmedabad, IND; 2 Spine Surgery, Bhavnagar Institute of Medical Science Hospital, Bhavnagar, IND

**Keywords:** bone tumor, cervical spine, enabling technology, en bloc resection, osteoblastoma, spinal neoplasms, surgical excision, transverse process, vertebral artery

## Abstract

Osteoblastoma is a rare benign bone tumor that may exhibit locally aggressive behavior. The cervical spine is the most frequently involved region, and delayed diagnosis can lead to progressive symptoms and neurological deficits. Surgical excision remains the definitive treatment to prevent morbidity. A 15-year-old male presented with a 14-month history of persistent neck pain and right upper limb radiculopathy. Radiological evaluation identified an expansile lesion arising from the right transverse process of the sixth cervical vertebra (C6), involving adjacent neurovascular structures. The lesion was surgically excised with meticulous preservation of the surrounding neurovascular anatomy. Intraoperative three-dimensional computed tomography (3D CT) was utilized to verify complete tumor excision, while continuous intraoperative neurophysiological monitoring (IONM) ensured neural function preservation and enhanced surgical safety. Histopathological examination confirmed osteoblastoma. Preoperatively, the patient exhibited severe disability (Oswestry Disability Index (ODI) score of 70) and intense pain (Numeric Rating Scale (NRS) score of 8). Postoperatively, remarkable clinical improvement was noted, with ODI reduced to 10 and NRS to 1, indicating minimal disability and significant pain relief. No recurrence was observed at five-year follow-up. This case highlights the crucial role of timely imaging and accurate diagnosis in cervical spinal osteoblastoma management. The combination of enabling technologies, namely intraoperative 3D CT and IONM, facilitates safe, complete tumor excision in vascularly complex locations and results in excellent functional recovery.

## Introduction

Osteoblastoma is a rare benign bone-forming tumor, accounting for less than 1% of all primary bone neoplasms [1]. It predominantly affects the spine, with approximately one-third of cases involving the cervical region [2]. Although histologically resembling osteoid osteoma, osteoblastoma can exhibit more aggressive behavior, leading to significant bone destruction and soft tissue invasion [3]. It typically presents in young adults during the second and third decades of life, with a higher incidence in males [4]. Clinical symptoms often include persistent, progressive neck or back pain, sometimes accompanied by radicular symptoms or neurological deficits due to compression of neural structures. Early diagnosis remains challenging because of vague and nonspecific symptoms; therefore, imaging modalities such as computed tomography (CT) and magnetic resonance imaging (MRI) are essential for accurate evaluation [5]. Complete surgical excision is critical to reduce the risk of recurrence, which occurs in 10%-20% of cases, mainly owing to incomplete resection [6].

This report highlights the rare presentation of a cervical osteoblastoma originating from the transverse process of the sixth cervical vertebra (C6) and demonstrates the successful surgical management supported by intraoperative three-dimensional CT and neurophysiological monitoring. By detailing this case, we aim to address the knowledge gap regarding surgical strategies in vascularly complex cervical osteoblastomas and emphasize the importance of integrating advanced intraoperative technologies to enhance resection completeness and patient safety.

## Case presentation

A 15-year-old male student presented with a 14-month history of progressively worsening right-sided neck pain radiating into the shoulder and upper arm. Pain was dull, persistent, worse at night, and partially relieved by analgesics. No history of trauma or constitutional symptoms was reported. Examination showed antalgic head tilt to the right, tenderness over the right lower cervical region, restricted neck movement, mild weakness in the right biceps and triceps, and paresthesia in C6 and cervical vertebra-7 (C7) dermatomal distribution. Pulses were symmetric with no cranial nerve or myelopathy signs.

**Figure 1:**
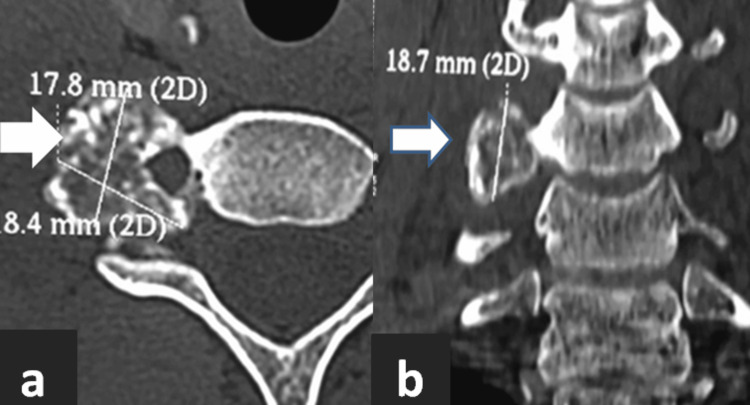
Preoperative CT showing a well-defined, lobulated, mixed lytic-sclerotic tumor mass measuring approximately 1.8 cm × 1.7 cm × 1.8 cm, originating from the right transverse process of the C6 vertebra. (a) Axial section CT image; (b) coronal section CT image CT: computed tomography; C6: sixth cervical vertebra

**Figure 2:**
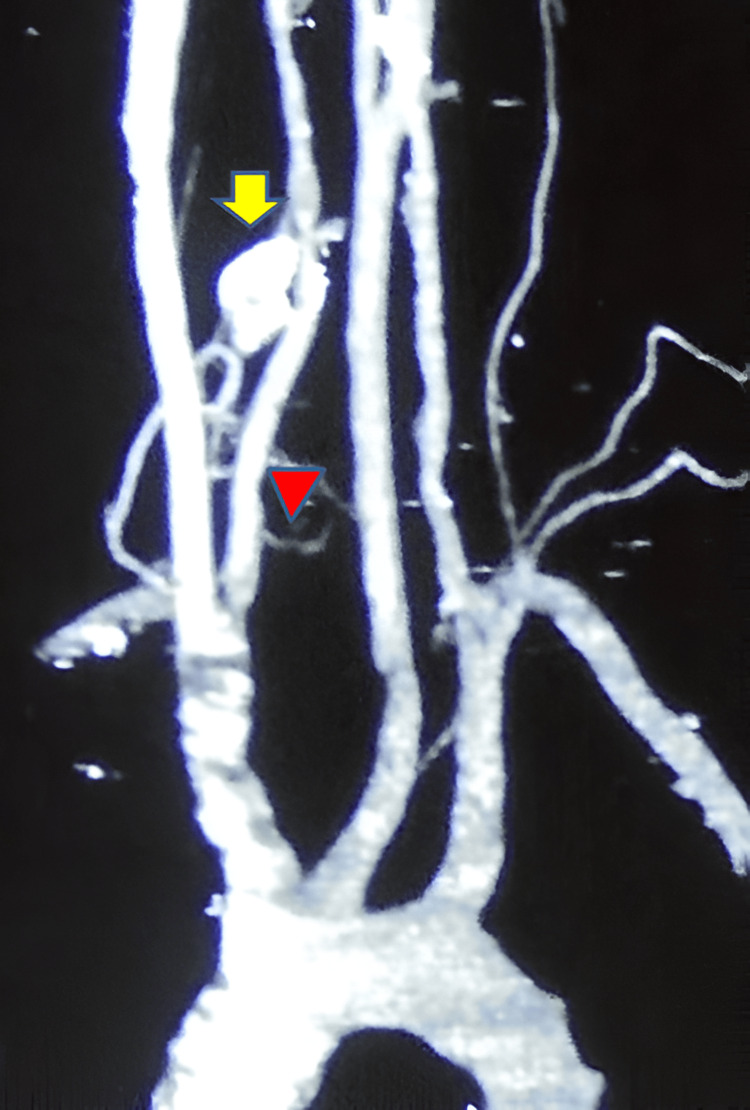
Three-dimensional reconstructed CT angiography with MIP image showing the tumor (arrow). The arrowhead indicates the right vertebral artery, which is encroached upon and displaced by the tumor, highlighting the critical vascular relationship important for surgical planning CT: computed tomography; MIP: maximum intensity projection

**Figure 3:**
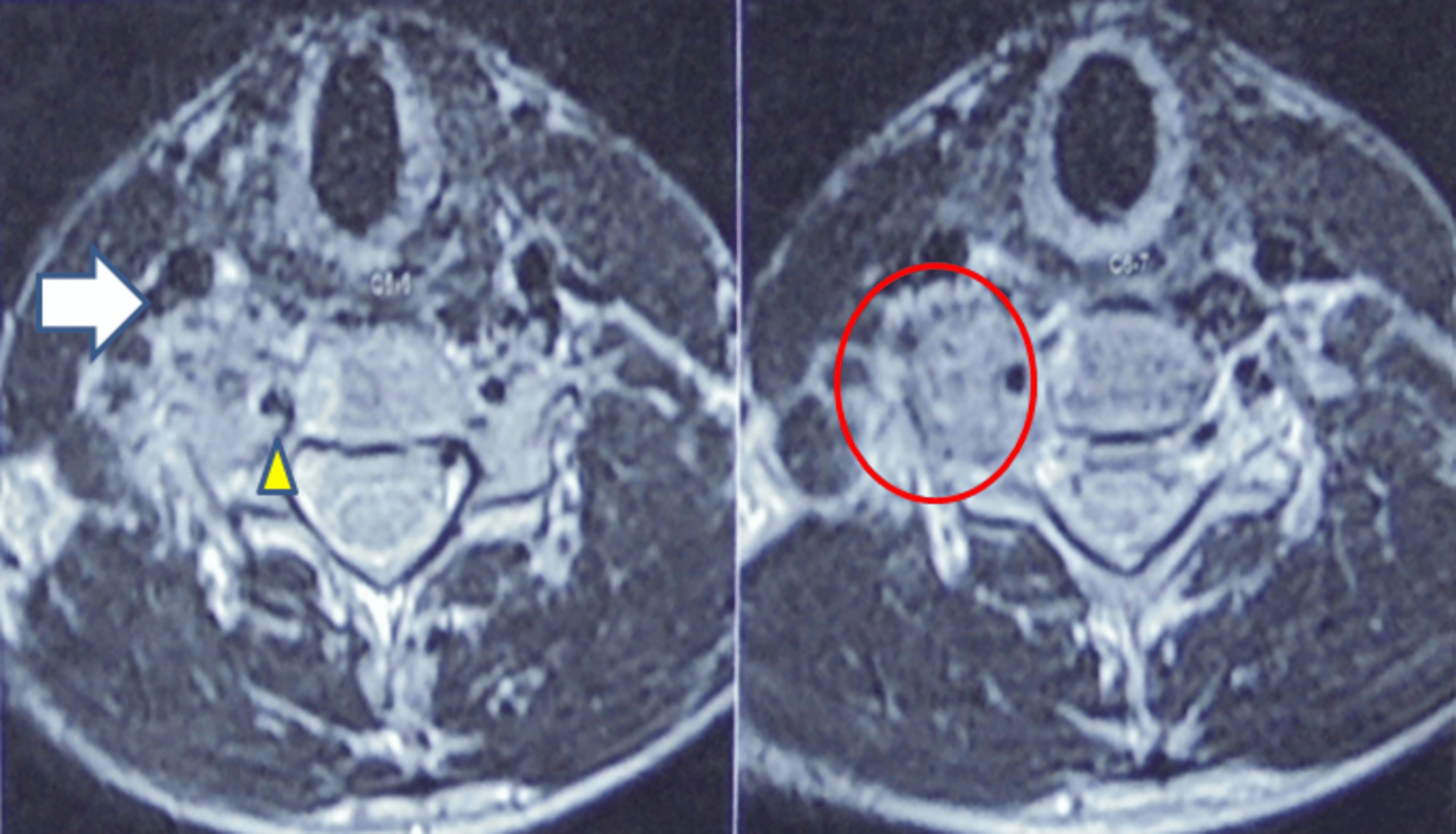
MRI of the cervical spine demonstrating tumor characteristics. The sequence shows heterogeneous intermediate to high intensity on T2-weighted images, consistent with a vascularized, mineralized lesion, which is marked in the circle. The arrow points to the proximity of the tumor to the right carotid artery, and an arrowhead points toward the right vertebral artery MRI: magnetic resonance imaging

Radiographic evaluation initially demonstrated a radiodense, well-demarcated lesion localized to the right C6 transverse process. This finding raised suspicion of a benign bone tumor given the sclerotic border. Advanced imaging with a CT scan revealed a well-defined, lobulated lesion measuring 1.8 cm × 1.7 cm × 1.8 cm exhibiting mixed lytic and sclerotic components as shown in Figure 1. The lesion involved the right C6 transverse process and facet joint, encasing but not compressing the ipsilateral vertebral artery. The lesion's mixed density pattern is characteristic of osteoblastoma, reflecting active osteoid matrix formation alongside cortical bone involvement. CT is essential to define the extent of bony destruction, lesion margins, and relationship to critical vascular structures, vital for surgical planning. CT angiography further confirmed patency of the vertebral artery with vascular encasement with no luminal compromise, as shown in Figure 2, highlighting the importance of preoperative vascular mapping in spinal osteoblastomas adjacent to major arteries. MRI sequences demonstrated heterogeneous intermediate to high signal intensity on T2-weighted images, consistent with vascularized tumor tissue interspersed with mineralized matrix, as shown in Figure 3. MRI is crucial to evaluate lesion extension into soft tissues, the spinal canal, neural foramen, nerve root involvement, and exclusion of cord compression. Knowledge of vertebral artery anatomy minimized intraoperative vascular injury risk.

The absence of periosteal reaction, cortical breach, or soft tissue mass on imaging reduced suspicion for an aggressive malignancy, supporting the diagnosis of benign osteoblastoma. Radiological differential diagnoses considered included aneurysmal bone cyst (typically fluid-fluid levels on MRI) and giant cell tumor (usually lytic without sclerotic margins).

Surgery was performed under general anesthesia with the patient positioned supine, ensuring optimal head stabilization and neck extension. The neck was carefully tilted to the left to provide maximal exposure of the right lower cervical region and to facilitate a safe surgical corridor to the lesion. After appropriate skin preparation and draping, a right-sided oblique cervical incision was made along the anterior border of the sternocleidomastoid muscle, following predefined anatomical landmarks to avoid injury to neurovascular structures.

**Figure 4:**
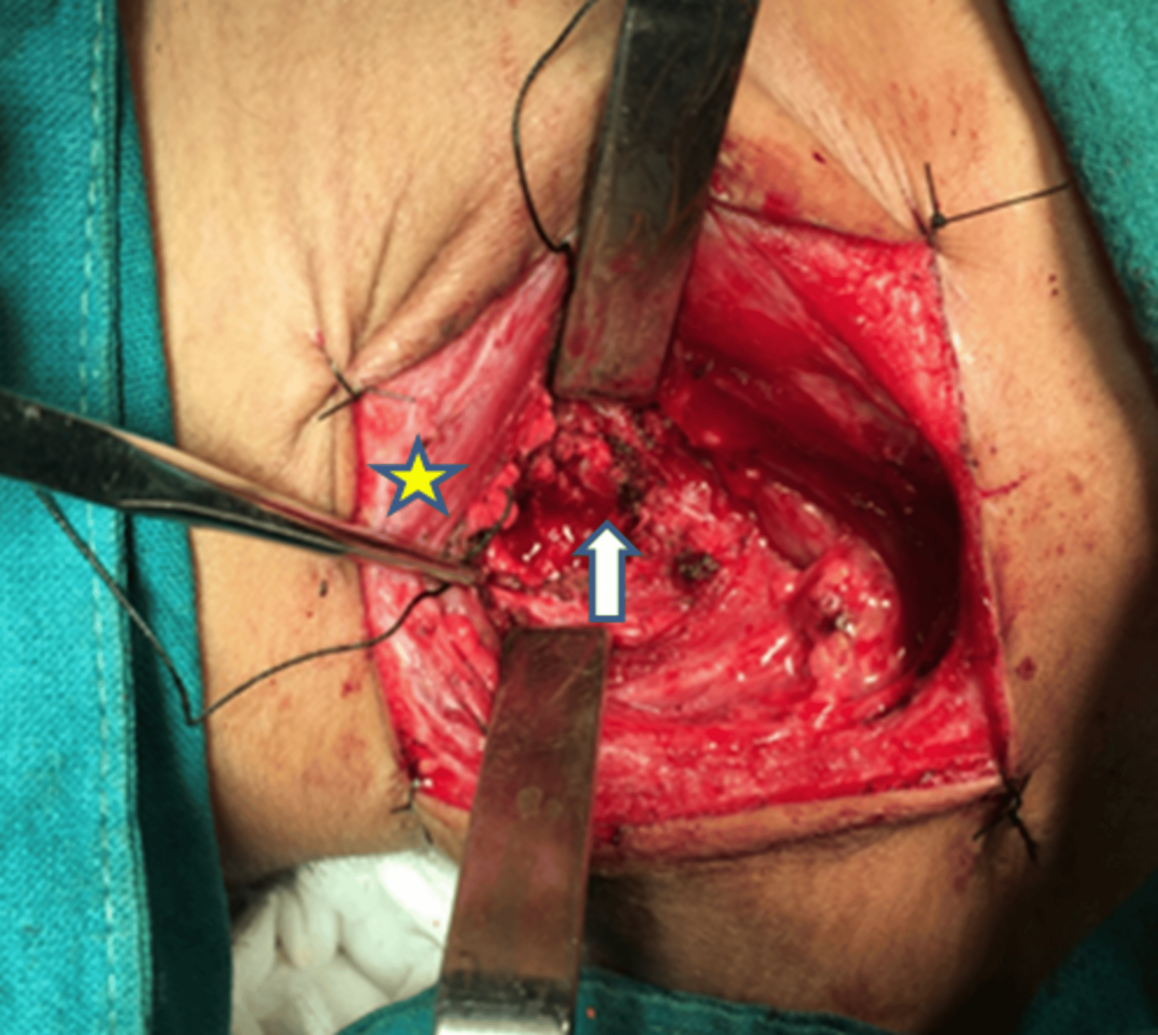
Intraoperative photograph showing exposure of the tumor during surgical resection. The star marks the sternocleidomastoid muscle retracted laterally to provide surgical access. The arrow points to the tumor mass

Using meticulous dissection techniques, the platysma was divided, and the plane anterior to the sternocleidomastoid was identified and developed. Careful blunt and sharp dissection exposed the prevertebral fascia and deeper layers, allowing identification of the tumor mass as shown in the intraoperative Figure 4, which was distinguishable from normal surrounding tissues by its firm, bony-hard consistency and increased vascularity.

The vertebral artery, critically adjacent to the lesion, was identified early in the procedure through anatomical landmarks and confirmed with microvascular techniques. Isolation of the vertebral artery was maintained throughout surgery by careful dissection of the surrounding soft tissue and by using vessel loops or retractors to prevent inadvertent injury.

Tumor removal was conducted in a piecemeal fashion to ensure maximal safe resection, facilitating control of vascular bleeding and minimizing manipulation of the artery and spinal nerves. C6 and C7 roots were identified. The right C6 facet and transverse process were partly removed. Hemostasis was achieved intermittently using bipolar cautery and hemostatic agents. Specimens were sent for histopathological evaluation to confirm the diagnosis.

Throughout the surgery, constant attention was given to maintaining the integrity of nearby nerve roots and spinal cord structures, guided by neuromonitoring when available, to avoid postoperative neurological deficits. Blood loss was minimal, approximately 100 mL, not necessitating transfusion, which is consistent with reported cases involving meticulous vascular control and careful surgical technique in highly vascular cervical osteoblastomas.

**Figure 5:**
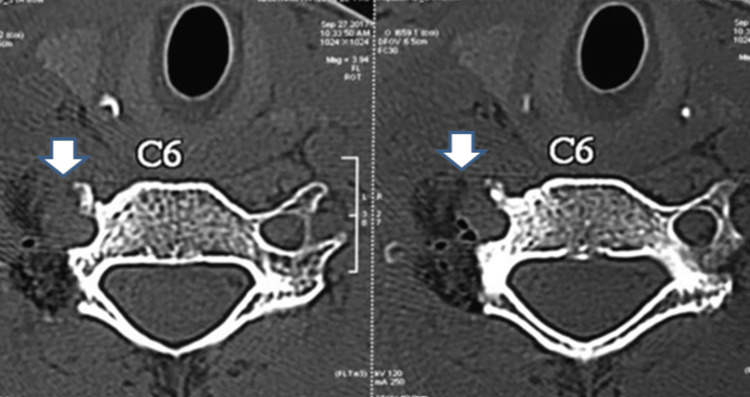
Intraoperative CT shows complete excision of the tumor, indicated by arrows CT: computed tomography; C6: sixth cervical vertebra

In this case, intraoperative three-dimensional computed tomography (3D CT) imaging played a pivotal role in confirming the completeness of tumor excision. After gross total resection, a 3D CT scan was performed intraoperatively to verify the absence of residual tumor and ensure adequate margins, as shown in Figure 5. This real-time imaging allowed for immediate correction if any tumor tissue remained and minimized the risk of local recurrence.

Intraoperative neurophysiological neuromonitoring (IONM) was utilized throughout the surgical procedure to safeguard neurological function. Continuous monitoring of motor and sensory pathways using techniques such as somatosensory and motor evoked potentials provided critical, real-time feedback on neural integrity. This allowed the surgical team to promptly detect impending nerve root or spinal cord injury and adjust maneuvers to prevent injury.

After a complete gross total resection was achieved, surgical site irrigation was performed, and meticulous layered closure was done. The patient was then extubated uneventfully and transferred to recovery for postoperative monitoring. The postoperative course was uneventful with immediate pain relief and mobilization on postoperative day one. No neurological or swallowing complications were noted. The patient was advised to use a soft neck collar for three weeks.

Preoperatively, the patient exhibited a high level of disability and pain, with an Oswestry Disability Index (ODI) score of 70, indicating severe functional impairment related to the cervical osteoblastoma [7]. The Numeric Rating Scale (NRS) pain score was 8, reflecting intense, persistent pain [8]. Following meticulous surgical resection, there was a marked improvement in the patient’s condition, with the ODI score decreasing significantly to 10 postoperatively, signifying minimal residual disability [7]. Correspondingly, the NRS pain score dropped to 1, indicating substantial pain relief [8].

**Figure 6:**
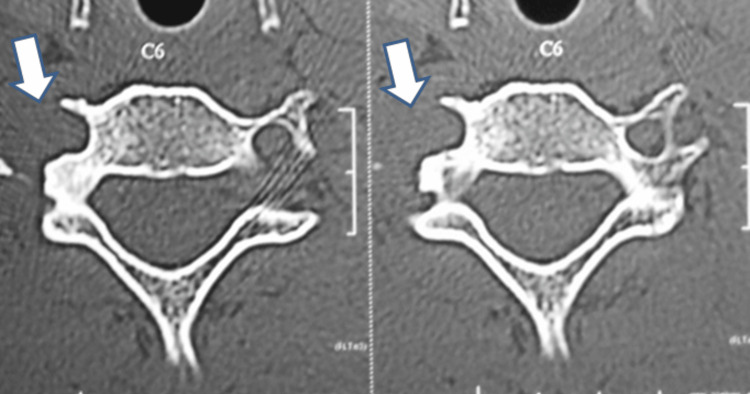
Postoperative axial CT scan at five-year follow-up showing total resection of the cervical osteoblastoma. Arrows indicate the surgical site, with no evidence of tumor recurrence CT: computed tomography; C6: sixth cervical vertebra

Follow-up evaluations at six months, two years, and five years confirmed sustained recovery without tumor recurrence, as shown in Figure 6, and complete neurological restoration.

## Discussion

Osteoblastomas affecting the cervical spine are a rare but distinctive type of benign bone tumor, known for their aggressive local behavior [1]. Their management poses considerable challenges, primarily due to the intricate anatomy of the cervical spine, which is closely associated with critical neural and vascular structures. The 15-year-old male patient described, who experienced persistent and progressive right-sided neck pain radiating to the shoulder and upper arm over 14 months, exhibited symptoms common in cervical osteoblastoma cases, underscoring the necessity for comprehensive clinical evaluation, precise imaging, and careful surgical planning.

Clinical presentation

The foremost symptom of cervical osteoblastoma is often persistent, dull neck pain that worsens progressively and can become resistant or only partially relieved by analgesics. Nighttime exacerbation of pain, together with neurological signs such as mild weakness in the C6-C7 myotomes and sensory disturbances, aligns well with findings from recent research where up to 70% of patients with cervical osteoblastomas report radiculopathy and neurological deficits [9]. Due to overlap with other cervical pathologies, early recognition of these progressive symptoms is vital to minimize diagnostic delay and facilitate timely intervention.

Radiological evaluation

Imaging plays a pivotal role in diagnosis and surgical planning. Plain radiographs may reveal radiodense lesions, while CT typically shows a well-circumscribed, lobulated lesion exhibiting a combination of lytic and sclerotic components characteristic of osteoblastoma. MRI is invaluable for assessing soft tissue extension and neural element involvement, although it may sometimes overstate tumor margins [10]. In cervical spine lesions, CT angiography assessment is crucial for evaluating the vertebral artery’s course and patency, thereby guiding surgical approach and minimizing intraoperative vascular injury [11]. Differential diagnoses such as aneurysmal bone cysts and giant cell tumors, which can present similarly in young patients, necessitate histopathological confirmation for definitive diagnosis.

Surgical considerations and tumor excision

Surgery remains the cornerstone of treatment for cervical osteoblastomas. The tumor’s proximity to vital structures like the spinal cord, nerve roots, and vertebral arteries complicates complete removal. Literature confirms recurrence rates of 10%-20%, predominantly linked to incomplete resections [6,12]. Although en bloc resection offers optimal local control, its feasibility is often limited in the cervical spine due to the adjacency to critical neurovascular structures [13]. The described piecemeal gross total resection with careful isolation of the vertebral artery exemplifies a balanced surgical approach to maximize tumor removal while maintaining spinal stability and vascular integrity. The reported modest blood loss mirrors outcomes in comparable cases, where smaller lesion size and meticulous technique reduce the need for transfusion [14].

The integration of enabling technologies within an Integrated Operation Theatre Spine Suite (IOTSS) facilitates safe and precise tumor resection by combining real-time intraoperative imaging, advanced navigation, and neuromonitoring. This coordinated setup enhances surgical accuracy, ensures preservation of critical neurovascular structures, and minimizes intraoperative complications, thereby improving overall clinical outcomes in complex spinal tumor surgery and helps in documentation also [15].

Intraoperative 3D CT imaging played a crucial role in this case by providing real-time verification of the extent of tumor resection. After gross total removal of the osteoblastoma, the intraoperative 3D CT scan allowed the surgical team to immediately confirm the absence of any residual tumor and ensure clear bony margins. This imaging modality is especially valuable because it offers precise anatomical detail during surgery, enabling prompt correction if any tumor remnants are detected. Such real-time feedback helps minimize the risk of local recurrence and improves the overall completeness of tumor excision, which is vital in spine tumor cases where reoperation can pose significant challenges [16].

In parallel, IONM was essential during the removal of the tumor near the C6 transverse process, providing continuous real-time feedback on the functional integrity of the nervous system. By utilizing somatosensory evoked potentials (SSEPs), the surgical team was able to monitor the sensory pathways, ensuring that stimulation of peripheral nerves was properly transmitted to the brain. Simultaneously, transcranial motor evoked potentials (TcMEPs) allowed assessment of motor pathways by recording muscle responses generated from direct stimulation of the motor cortex, giving immediate insight into the status of the corticospinal tracts. This dual monitoring was critical, given the tumor’s proximity to vital nerve roots and the vertebral artery. It enabled the team to detect early warning signs of neural distress, such as changes in signal amplitude or latency, and to adapt surgical maneuvers accordingly to avoid permanent damage. The combination of 3D CT imaging with IONM ensures a comprehensive approach to maximize tumor resection while preserving neurological function, reflecting advanced standards in neurosurgical tumor management [17].

Role of preoperative embolization

Osteoblastomas are known for their hypervascularity, which can cause significant intraoperative bleeding. Preoperative arterial embolization is recognized as an effective adjunct to decrease intraoperative blood loss and facilitate safer resections, especially for large or highly vascular lesions. However, in smaller, less vascular tumors such as in this patient, embolization was not necessary [18].

Postoperative outcomes and follow-up

Preoperatively, the patient exhibited a high level of disability and pain, with an ODI score of 70, indicating severe functional impairment related to the cervical osteoblastoma [7]. The NRS pain score was 8, reflecting intense, persistent pain [8]. Following meticulous surgical resection, there was a marked improvement in the patient’s condition, with the ODI score decreasing significantly to 10 postoperatively, signifying minimal residual disability. Correspondingly, the NRS pain score dropped to 1, indicating substantial pain relief. These postoperative scores underscore the effectiveness of aggressive but careful surgical management in alleviating symptoms and restoring function in patients with cervical spine osteoblastoma, consistent with contemporary clinical outcomes reported in recent literature [10]. This improvement aligns with favorable neurological recovery and significantly enhanced quality of life after surgery. Follow-ups at six months and two years showed no recurrence and complete neurological recovery.

The patient’s rapid postoperative pain relief, early mobilization, and sustained two-year recurrence-free survival with full neurological recovery demonstrate the success of meticulous surgical planning and execution. Long-term follow-up remains critical to identify any signs of tumor recurrence or rare malignant transformation early.

Adjuvant treatments with radiotherapy and radiofrequency ablation

Radiotherapy’s role is limited due to concerns regarding malignant transformation and is typically reserved for lesions that are unresectable or recurrent [9]. Radiofrequency ablation (RFA), effective mainly in smaller osteoid osteomas, is not widely recommended for cervical osteoblastomas because of the risk of thermal injury to adjacent neurovascular structures [19,20]. Thus, adjuvant therapies serve a complementary rather than primary role in managing cervical osteoblastomas.

This case highlights the challenges involved in managing cervical osteoblastomas, particularly due to their close proximity to critical neurovascular structures. The combined application of intraoperative 3D CT imaging and continuous neuromonitoring substantially improves both the precision and safety of tumor removal in such complex scenarios. Compared to previous reports, our approach provides enhanced confidence in achieving complete resection while preserving neurological function, thereby potentially reducing recurrence rates. Given these advantages, we advocate for the routine integration of these advanced intraoperative technologies in surgeries for spinal tumors characterized by vascular complexity to optimize patient outcomes and surgical success.

## Conclusions

This case highlights the inherent complexities in managing cervical osteoblastomas, which arise from the dual challenge of aggressive tumor biology and the delicate anatomy of the cervical spine. Current literature emphasizes the importance of comprehensive multimodal imaging, including CT, MRI, and angiography, to guide meticulous surgical planning that seeks to achieve complete excision while preserving critical structures. The integration of enabling technologies within an IOTSS facilitates safe and secure tumor resection, with intraoperative 3D CT imaging providing real-time confirmation of excision. Additionally, the use of continuous IONM serves as a safeguard for neurological integrity, together ensuring precise tumor removal and optimal surgical outcomes.
